# Precision Agriculture Applied to Harvesting Operations through the Exploitation of Numerical Simulation

**DOI:** 10.3390/s24041214

**Published:** 2024-02-14

**Authors:** Federico Cheli, Ahmed Khaled Mohamed Abdelaziz, Stefano Arrigoni, Francesco Paparazzo, Marco Pezzola

**Affiliations:** 1 Department of Mechanical Engineering, Politecnico di Milano, Via La Masa 1, 20156 Milano, Italy; federico.cheli@polimi.it (F.C.); stefano.arrigoni@polimi.it (S.A.); 2 Soluzioni Ingegneria, Via Lorenzo Balicco 113, 23900 Lecco, Italy; ahmedkhaled.abdelaziz@mail.polimi.it (A.K.M.A.);

**Keywords:** precision agriculture, harvesting, path planning, path tracking, CarMaker, agricultural simulator

## Abstract

When it comes to harvesting operations, precision agriculture needs to consider both combine harvester technology and the precise execution of the process to eliminate harvest losses and minimize out-of-work time. This work aims to propose a complete control framework defined by a two-layer-based algorithm and a simulation environment suitable for quantitative harvest loss, time, and consumption analyses. In detail, the path-planning layer shows suitable harvesting techniques considering field boundaries and irregularities, while the path-tracking layer presents a vision-guided Stanley Lateral Controller. In order to validate the developed control framework, challenging driving scenarios were created using IPG-CarMaker software to emulate wheat harvesting operations. Results showed the effectiveness of the designed controller to follow the reference trajectory under regular field conditions with zero harvest waste and minimum out-of-work time. Whereas, in presence of harsh road irregularities, the reference trajectory should be re-planned by either selecting an alternative harvesting method or overlapping the harvester header by some distance to avoid missing crops. Quantitative and qualitative comparisons between the two harvesting techniques as well as a relationship between the level of irregularities and the required overlap will be presented. Eventually, a Driver-in-the-loop (DIL) framework is proposed as a methodology to compare human and autonomous driving.

## 1. Introduction

This paper aims to review the previous work and research developments in the field of autonomous combine harvesters and precision agriculture from the point of view of simulation scenarios, cutting methods, control architectures, and sensors.

The integration of crop growth simulation has become integral in advancing the understanding of agricultural systems. Some of the most prominent models includes APES, CROPSYST, DAISY, DSSAT, FASSET, HERMES, STICS, and WOFOST, which integrate daily weather statistics, soil properties, and detailed crop and soil management information. The study conducted in [1] serves as an important reference, offering a comparative analysis of these models in simulating winter wheat yield and variability across different climates in Europe. The comprehensive use of daily weather statistics, soil properties, crop phenology, and management information in these models reflects their capacity to significantly contribute to the advancement of agricultural science.

**Figure 1:**
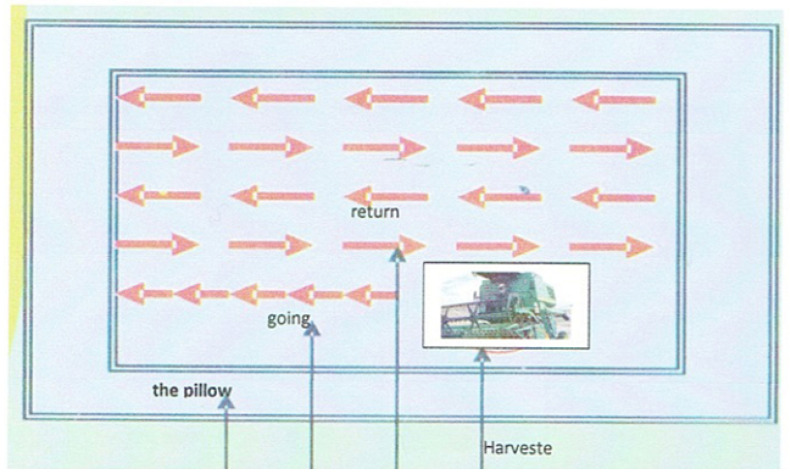
Harvesting Method by Going and Return, source [2].

**Figure 2:**
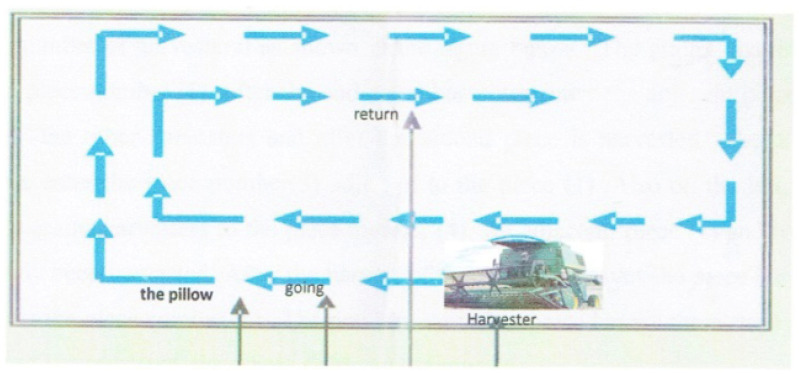
Harvesting Method by Rotation Oceanic, source [2].

In the context of wheat harvesting, the choice of cutting technique significantly influences the overall effectiveness of the process and has a direct impact on harvest losses. Two notable harvesting techniques, “Harvesting Method by Going and Return” shown in Figure 1 and “Harvesting Method by Rotation Oceanic” shown in Figure 2 have been explored in the literature to address the challenge of minimizing harvest losses [2].

**Figure 3:**
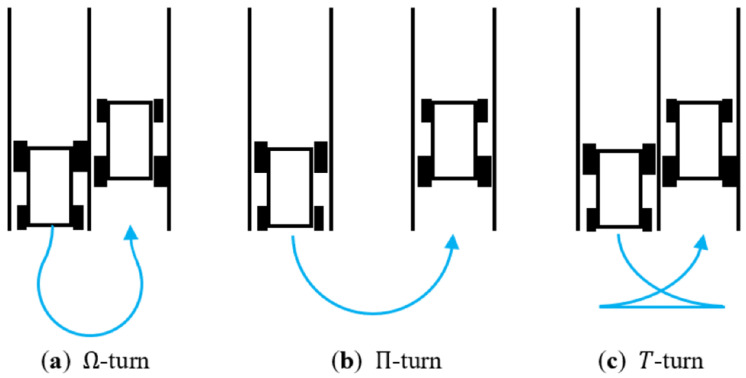
Three commonly used maneuvers in the headland turns, source [4].

In both of the approaches, efficient headland (end-of-row) maneuvering stands as a critical element for operational effectiveness, as shown in Figure 3. While automatic guidance systems commonly excel in parallel tracking, the inclusion of headland maneuvering is often overlooked. The conventional Dubins Curves method, employed for path generation, lacks consideration for critical parameters like maximum steering angle [3]. Addressing this gap, an algorithm was introduced to formulate a seamless path for headland maneuvering, ensuring continuity in curvature and speed. Notably, the algorithm incorporated considerations for both the maximum steering angle and the maximum acceleration of the agricultural vehicle. Moreover, it allowed for the definition of a target speed profile, offering the flexibility to either increase or decrease the speed during the turn. It also enabled the smooth connection from zero curvature to maximal curvature, which represents the reciprocal of the minimum turning radius [4].

**Figure 4:**
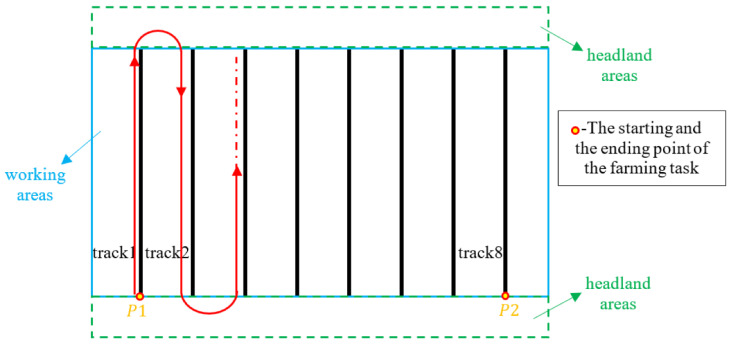
The given optimized tracking trajectory by the operational layer (red curves), source [4].

In the field of agricultural automation, the primary focus of path-tracking control algorithms is often directed towards enhancing the trajectory-tracking performance of autonomous agricultural machinery. Typically, agricultural productivity is optimized through a two-layer approach. The upper operational layer strives to optimize an ideal tracking trajectory that maximizes productivity; see Figure 4. Meanwhile, the lower control layer is responsible for receiving this optimized trajectory and steering the vehicle with high precision to follow it [5].

Central to this endeavor is the design of path-tracking controllers that carefully consider the stability of the yaw (heading angle) and the constraints inherent in vehicle dynamics. Current research predominantly revolves around a lateral controller design, often relying on the identification of a path reference point based on the shortest distance to the vehicle from the current state. However, this approach imposes limitations on the controller’s adaptability to sudden changes in the trajectory heading angle [6]. In [7], a novel approach to path tracking aimed to emulate human driving behavior. Departing from traditional methods, this approach was grounded in a discrete prediction model that anticipates the future states of the vehicle. By enabling the control algorithm to operate on future-predicted states in conjunction with the current controller output, the proposed methodology sought to enhance the system’s responsiveness to dynamic changes in the environment. In contrast, the work presented in [8] mainly aimed at automatizing the U-turn of combine harvesters to account for both vehicle constraints and overlap in order to eliminate harvest losses. In the context of harvesting, overlap refers to the repeated coverage of a portion of the field or crop by the combine header during successive passes. Overlap can occur when the harvesting equipment covers more ground than necessary, resulting in redundant or double harvesting of certain areas. In understanding harvest losses, several factors play a crucial role; however, in this project the focus is only on the field topography effect on harvest losses in the context of precision agriculture.

To efficiently address the soil irregularities, the choice of a proper sensor is crucial in order to obtain a reliable feedback. For this reason, sensor-based controllers play an important role in autonomous agriculture. Recently, [9] introduced a noteworthy technological advancement in wheat harvesting, enhancing the efficiency of the harvesting process by integrating a GPS receiver and a grain level sensor into a wheat harvester combine. In [10], the authors introduced an approach that aimed to address the specific needs of small-scale wheat farmers by developing a scalable, low-cost technique for wheat harvesting. The core of their methodology involved the integration of a standard camera, in conjunction with a GPS-assisted unit, served as the sensory input for the system. An image-processing module was employed to extract relevant information from the captured images, with data sourced from the sensor module. A microcontroller was responsible for steering and driving motor control, allowing the robot to navigate the wheat fields effectively.

## 2. Motivations and Methodology

The motivation behind research in autonomous agriculture stems from addressing various challenges in traditional harvesting methods. They include the following:

- Operator Stress and Fatigue: the current reliance on manual operation leads to operator stress and fatigue, affecting overall efficiency and well-being;
- Challenging Field Conditions: conventional harvesting faces difficulties in effectively navigating and operating in challenging field conditions;
- Soil Compaction and Passes: manual harvesting can contribute to soil compaction due to excessive passes, impacting soil health and overall crop yield;
- Damaged or Missing Crops: human-operated harvesters may inadvertently cause crop damage during the harvesting process, leading to yield losses;
- Tire and Track Wear: traditional harvesting equipment experiences wear and tear on tires and tracks, requiring frequent maintenance and replacement;
- High Fuel Consumption: manual harvesting often involves high fuel consumption, contributing to increased operational costs and environmental concerns [11].

To test a wide variety of scenarios, especially regarding soil irregularities, the entire work is conducted in simulation. This paper is structured as follows. In Section 3, a proper scenario and harvester modeling is presented. This work makes use of IPG-CarMaker software to create wheat crops and soil, import a harvester model within the scenario, and then perform virtual driving tests to model wheat harvesting operations and validate autonomous driving control algorithms. In Section 4, vehicle modeling is reported. In Section 5, path-planning and path-following algorithms are described. This work presents an effective Stanley Lateral Controller developed on Simulink and tested on CarMaker, for either tracking a predefined trajectory assuming knowledge of the agricultural field or following the field borders online by means of a vision sensor for crop edge guidance. In Section 6, a quantitative comparison between two harvesting techniques—cutting by go-and-return and oceanic cutting—in terms of harvesting time, fuel consumption, and need for overlapping the cutter to eliminate harvest losses is presented. Moreover, a framework to compare manual driving and automated driving is proposed. Eventually, in Section 7, conclusions are drawn based on the previously listed motivations.

## 3. Scenario Generation

### 3.1. Environment Definition

**Figure 5:**
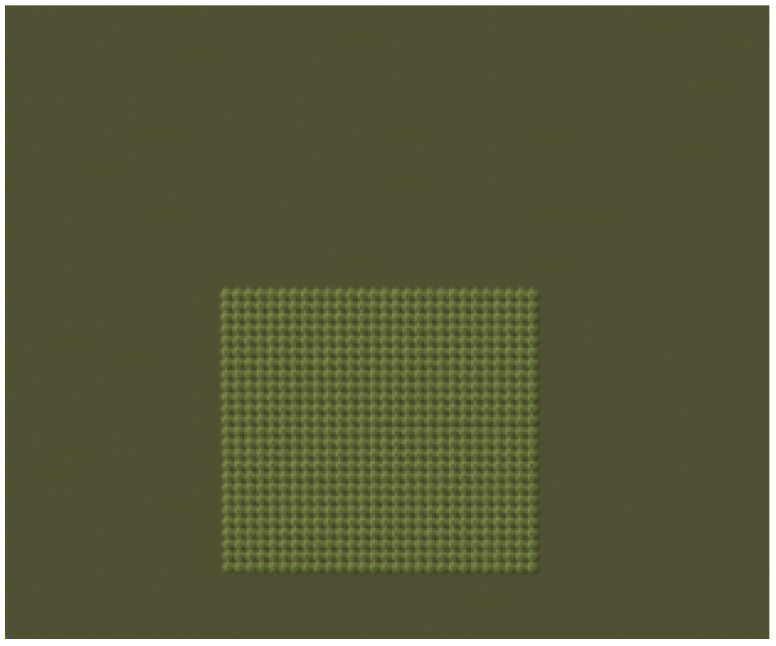
Wheat field model.

To model a wheat field, the following scenario was created through the editor tab of CarMaker [12]: a vast space of land (road) where a 50-square-meter dense area of wheat crops was added. Crops were modeled as traffic objects of type ‘Bush’; see Figure 5.

**Figure 6:**
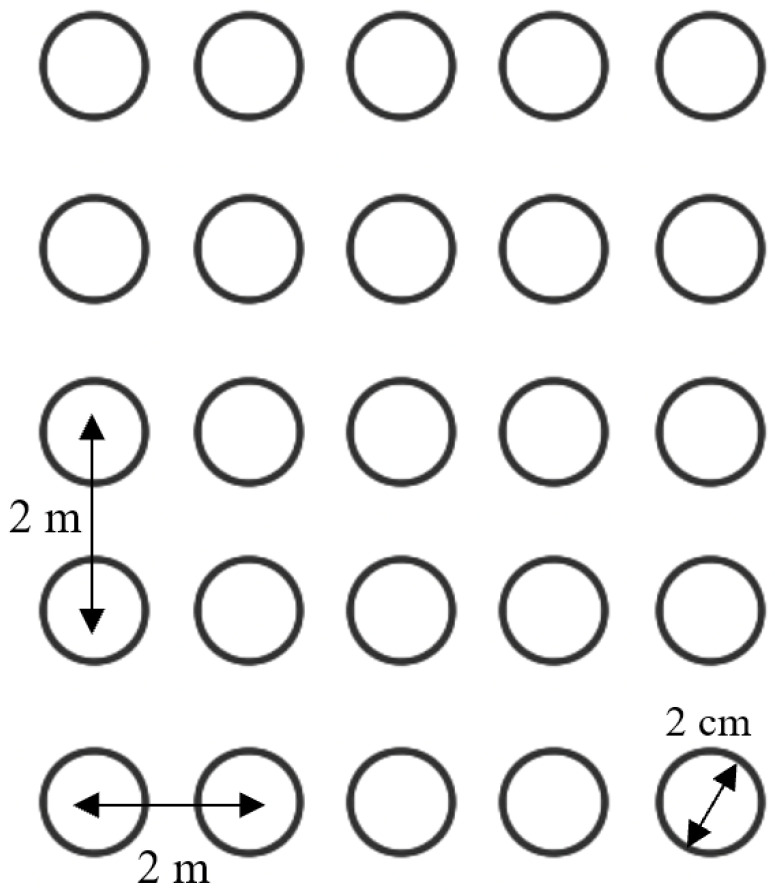
Harvester-based crop model.

As the number of traffic objects in the scenario was limited to 700, the crops were 2 m apart from each other to make a relatively large but dense field. However, to have a realistic model of the crop size, virtual 1-centimeter-radius circles were placed around the center of each crop by means of C code. Thus, from a vision sensor point of view, that area was fully dense of quite big crops, while from the harvester point of view, the crops were small and distant, as shown in Figure 6.

**Figure 7:**
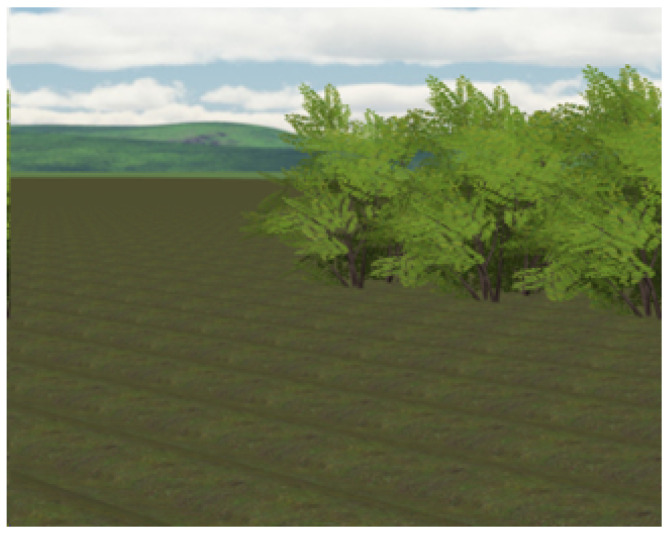
Wavy field.

Eventually, to make this scenario resemble a challenging wheat field, irregularities were introduced through wave function of different orientations, heights, and period lengths. A schematic representation is shown in Figure 7.

### 3.2. Harvest Modeling

**Figure 8:**
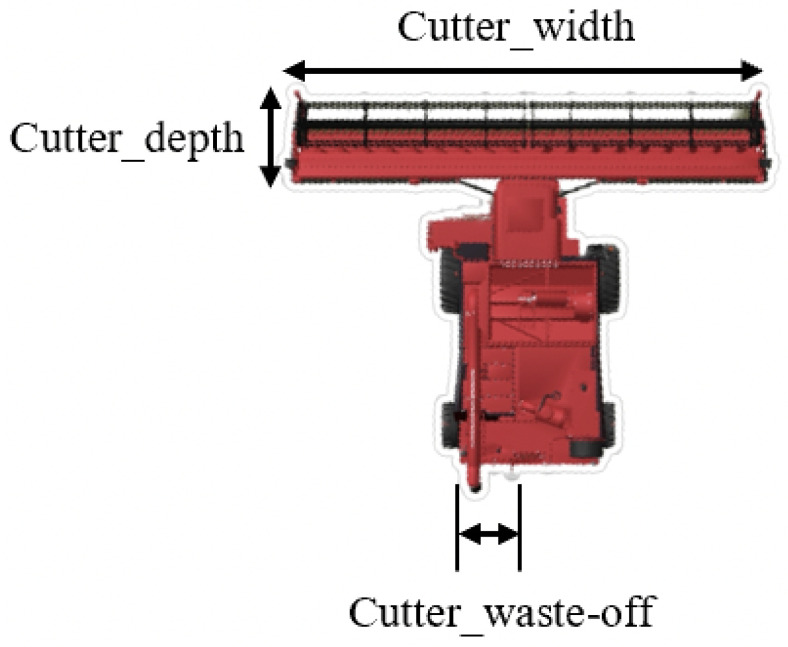
Generic harvester.

A combine harvester is a machine that is primarily used for harvesting cereal crops such as wheat. It is designed to cut, thresh, and clean the crop in one operation, separating the grain from the straw and chaff. The grain is collected in a tank for transport, while the straw is either spread back onto the field or baled for use as animal feed or bedding. As an example, John Deere X9 1100 stands as a cutting-edge combine harvester ranking among the largest globally [13]. A generic combine harvester model was first defined based on four parameters, namely, cutter width, cutter depth, cutter waste-off, and cutter waste rate. For details, see Figure 8.

**Figure 9:**
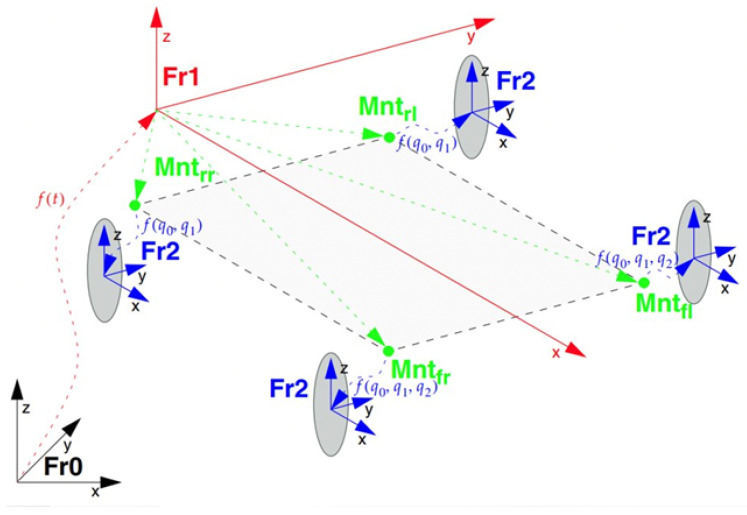
Combine’s pose in the global reference frame, source [14].

While the cutter waste-off and waste rate were additional parameters for determining where and how often to place waste behind the combine, the cutter width and depth were fundamental in the cutting mechanism. The cutter’s end line shown in Figure 8 was modeled as a virtual moving segment of length equal to the cutter width, slope equal to the combine heading angle (ψ), and intercept depending on the position of the combine reference frame (Fr1) in the global one (Fr0). It was developed as a custom C code for the specific application. The concept is easily explained by Figure 9 and Equation (1) [14].

The translation matrix (t) and rotation matrix (r) from Fr0 to the end points of the cutter’s virtual line are defined as follows:

(1) $$\left\{ \begin{array}{l} t=\left[\begin{array}{c} X+l+cutter\;depth\\ Y\pm \frac{\mathrm{cutter}\;\mathrm{width}}{2}\\ 0 \end{array}\right]\\ r=\left[\begin{array}{ccc} cos\;\psi & -sin\;\psi & 0\\ sin\;\psi & cos\;\psi & 0\\ 0 & 0 & 1 \end{array}\right] \end{array}\right.$$

**Figure 10:**
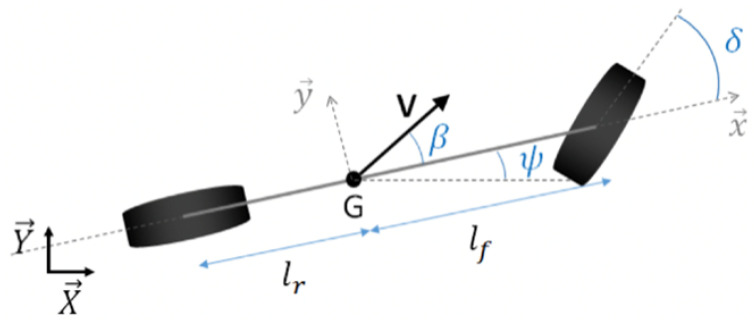
Vehicle kinematic model, source [15].

As shown in Figure 10, l is the combine length in meters, and X-Y (meters) and ψ (radians) characterize the pose of Fr1 in the global frame Fr0, which are obtained directly as accessible quantities from the simulation environment.

**Figure 11:**
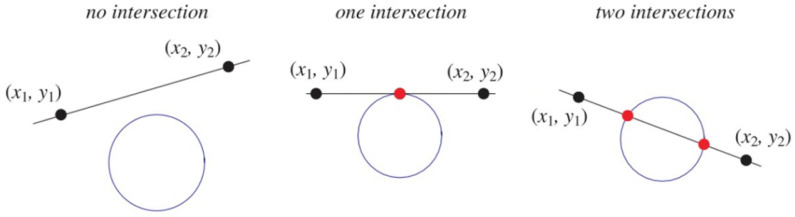
Cutting condition by the intersection of a line and circle.

Once the dynamic virtual line intersects at least one time with with any of the fixed virtual circles previously defined around the center of each crop (this is detected by a custom routine defined) as reported in Figure 11, the corresponding crop disappears from the scenario emulating a real harvesting operation.

## 4. Vehicle Model

**Figure 12:**
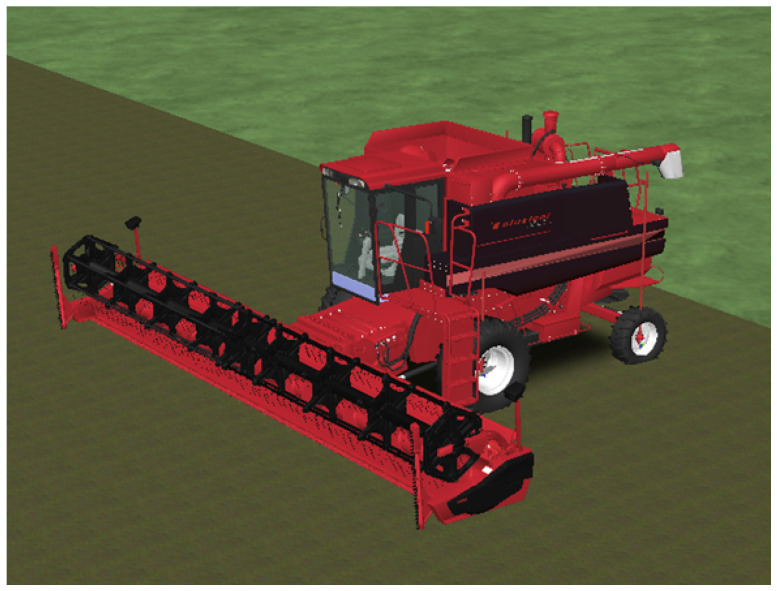
Harvester 3D object.

In order to move from a generic model to a more appropriate one capable of taking into account kinematic and dynamic characteristics of a real harvester, a 3D model of the harvester was included in the virtual environment, as shown in Figure 12.

Agricultural machinery should be modeled with high-fidelity nonlinear dynamic models, in order to highlight and consider every force that affects the system. However, this approach would require an extensive modeling of the environments (for example, slipping effects, which depend on soil moisture and tire properties) and a high computational burden. For this reason, under the hypothesis of low harvesting speeds, a kinematic modeling was considered a good compromise for the study. The scheme is reported in Figure 10, where $l_{r}$ and $l_{f}$ represent the longitudinal distances from center of gravity (G) to the rear and front axles, respectively, v is the velocity vector, β is the slip angle and is assumed to be zero, and δ is the steering angle.

The mathematical formulation of the kinematic model, as well as system states (z) and control input (u), are defined in Equation (2), respectively.

(2) $$\left\{ \begin{array}{l} \left[\begin{array}{c} \dot{X}\\ \dot{Y}\\ \dot{\psi } \end{array}\right]=\left[\begin{array}{c} v\cdot cos\;\psi \\ v\cdot sin\;\psi \\ \frac{v\cdot \tan\;\delta }{l_{r}+l_{f}} \end{array}\right]\\ \;\;z=\left[\begin{array}{c} X\\ Y\\ \psi \end{array}\right]\\ \;\;u=\delta \end{array}\right.$$

## 5. Control Architecture

### 5.1. Path Planning

**Figure 13:**
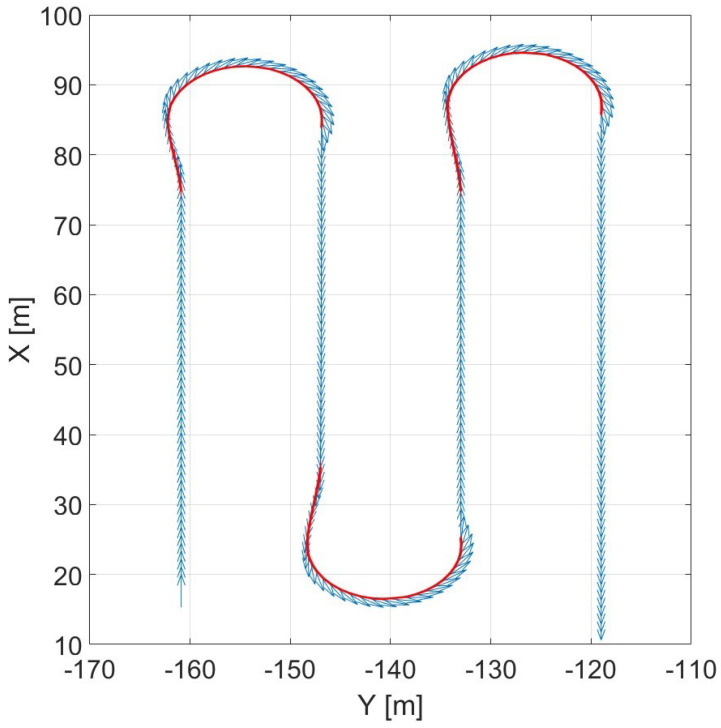
Vehicle trajectory in cutting by go-and-return.

Cutting by go-and-return is the most popular harvesting technique and the easiest to control. Its most challenging sub-task consists in the planning phase of the $180^{o}$ end-of-row turn, highlighted in red in Figure 13. The advantage of this techniques is represented by the combine’s ability to demonstrate an almost constant speed in the following trajectory performed.

These turns, however, need to minimize the out-of-work time while considering the vehicle constraints: maximum and minimum speed in [m/s] and maximum steering angle in [rad]. In order to minimize distance traveled from an initial pose characterized by $0^{o}$ or $180^{o}$ heading angle to a final pose characterized by $180^{o}$ or $0^{o}$ heading angle, an optimization problem was formulated, as shown in Equation (3) and numerically solved offline (MATLAB routine).

(3) $$\left\{ \begin{array}{c} \min J=\sum_{j=1}^{N}({\left\|z_{i}-z_{i-1}\right\|}^{2}\cdot \alpha )\\ s.t.z(0)=z_{0},\\ z(N)=z_{1},\\ v_{\min}≦v_{i}≦v_{\max}\\ |\delta_{i}|≦\delta_{\max} \end{array}\right.$$

In the above, J is the cost function to be minimized, i.e., the sum of the squared distances between two consecutive points in the trajectory. N is the total number of (spatial) discretization points of the trajectory (for the considered case, N is set to be equal to 20), i is the index of a point in the trajectory, (α) is a weighting matrix, and $z_{0}$ and $z_{1}$ are the initial and final poses, respectively.

**Figure 14:**
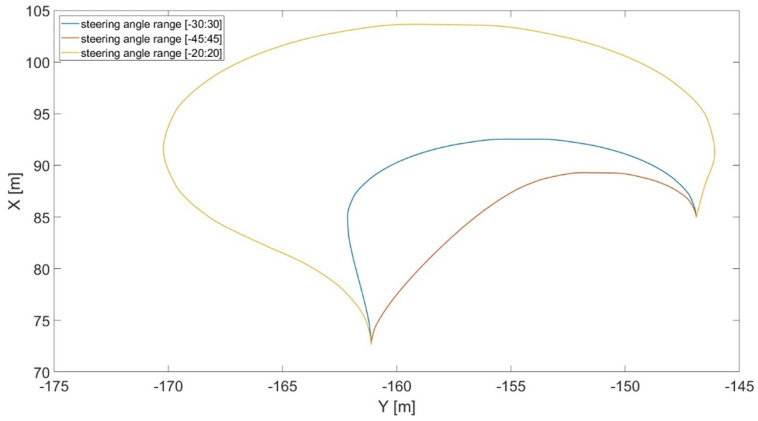
Effect of the steering angle range constraint (in degrees) on the optimal trajectory.

In Figure 14, steering angle range constraint influence over the resulting maneuver is presented as an example.

In detail, narrowing the steering angle range leads to longer travel distances and more out-of-work time; furthermore, increasing the speed minimum value beyond 20 km/h makes the vehicle unable to take this sharp turn. Hence, suitable ranges for both steering angle and velocity were selected as [−30:30] degrees and [1:20] km/h according to the numerical results. Moreover, these values are consistent with the physical constraints of the typical state-of-the-art combine harvesters.

**Figure 15:**
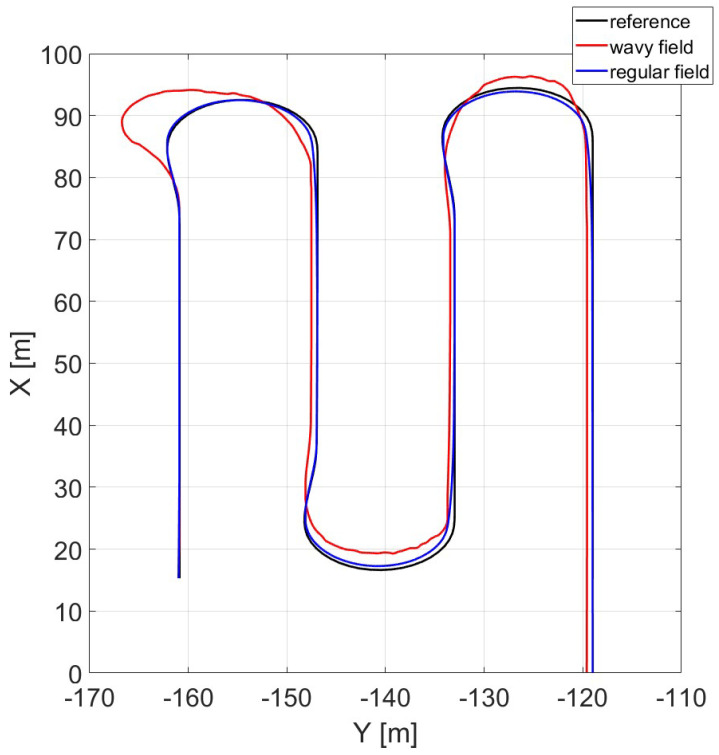
Reference vs. actual trajectories in wavy and regular fields.

Irregularities are introduced in the virtual environment in order to reproduce a more realistic scenario and verify tracking performances of the control system presented. A comparison between regular field (blue) and wavy field (red) tracking results is presented in Figure 15, where the effect is well represented. In detail, the wavy field scenario considers a wave orientation equal to $90^{o}$, wave height equal to 20 cm, and wave period length equal to 2 m.

**Figure 16:**
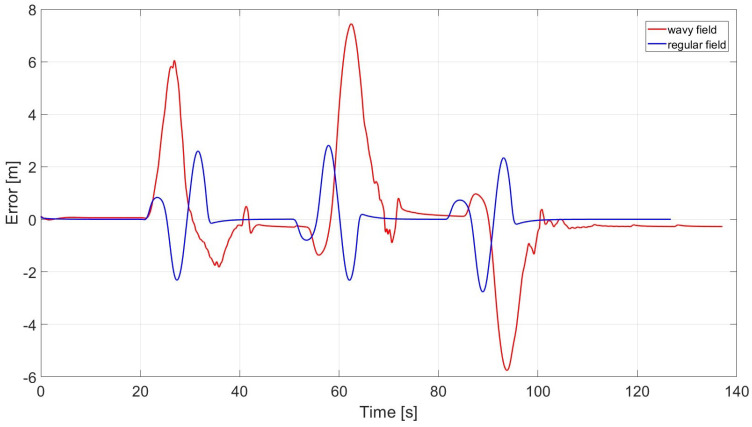
Errors in wavy and regular fields.

The error (lateral deviation from ref.) associated with each of the two cases is shown in Figure 16.

As can be seen from Figure 15 and Figure 16, the lateral deviation from the reference trajectory in the case of a wavy field is not negligible, and will inevitably lead to harvest losses. This leads to the necessity of adopting a safe overlap distance (at least equal to the maximum value of error) inside the field. This affects the optimal turn numerical calculation where the considered width becomes equal to the difference between the cutter depth and the overlap. However, this implies that the combine has to pass twice through a certain area at the intersection of two rows, which might cause soil compaction.

**Figure 17:**
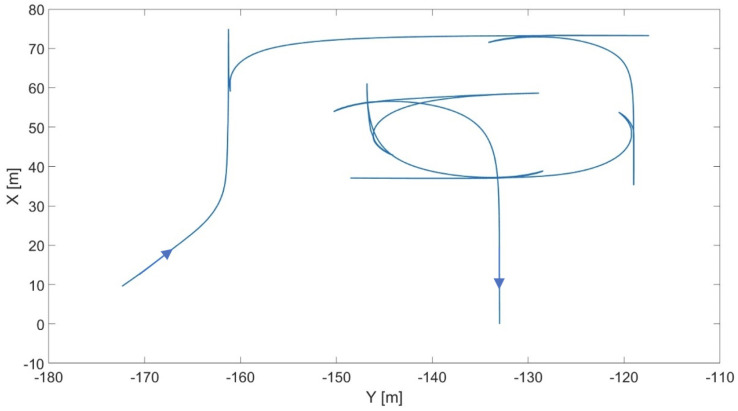
Vehicle trajectory in oceanic cutting.

An alternative solution is to adopt a completely different harvesting technique called oceanic cutting, which has the advantages of reducing harvest losses to zero without the necessity of overlap and self-containing the combine within the field’s borders (which may be important in case it is not possible to perform a U-turn due to space constraints). However, this technique presents some known drawbacks such as a longer harvesting time, more fuel consumption, and ride discomfort because of the reverse motion direction required at the end of each row/column performed. An example of trajectory performed during oceanic cutting is shown in Figure 17.

### 5.2. Path Tracking

In order to properly track the reference trajectory computed by each harvesting technique, a Stanley Lateral Controller was implemented. This path-tracking technique was firstly introduced by Standford University’s Darpa Grand Challenge team as reported in [16]. Unlike other path-tracking methods, which make use of the rear axle as reference point, Stanley methods use the front axle. The controller is specifically designed in order to minimize both the heading error and cross-track error while ensuring the steering angle to remain in a feasible range of motion. As a result of this control architecture, steering input equations are reported in Equation (4):

(4) $$\left\{\begin{array}{c} \delta \left(t\right)=\psi \left(t\right)+\tan^{-1}\left(\frac{k\cdot e(t)}{k_{s}+v\left(t\right)}\right)\\ \;s.t.\;\delta \left(t\right)\in \left[\delta_{\min},\delta_{\max}\right] \end{array}\right.$$

where k is the position gain of forward motion, $k_{s}$ is a softening constant to ensure the denominator is non-zero, e(t) is the cross-track error, and ψ is the heading error. The Cross-track error is defined as the distance between the closest point on the path with the front axle of the vehicle (lateral deviation seen in Figure 16) while heading error is the difference between heading angle and trajectory angle. Both errors are evaluated according to path references defined in Figure 13 and Figure 17 for the different cutting methods.

In this paper, path tracking is computed by means of the use of GNSS data and by means of a distance sensor-based data.

In the first implementation presented, GNSS data are considered as measurement variables. In this case, the procedure directly computes tracking, comparing measured position and orientation to the reference path previously computed offline. This approach is required when local measurements (distance sensors or cameras) are not available, but accuracy may be reduced according to weather conditions and GNSS reliability.

**Figure 18:**
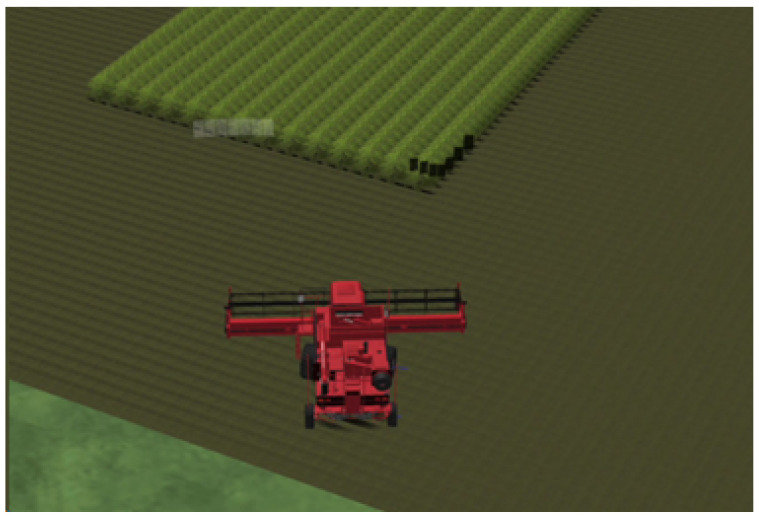
Free Space Sensor detection.

For this reason, a more reliable approach based on local measurements was developed. In detail, the possibility of taking advantage of relative distance information between the vehicle and crops was analyzed. Considering CarMaker virtual simulator software, the approach developed is based on the use of two “Free Space” sensors mounted on the most right and left positions of the cutter. The “Free Space” sensor is an ideal sensor module used to scan the environment like a stereo camera and capture the free and the occupied space around the vehicle, as shown in Figure 18.

The design choice adopted consists of placing the harvester at any arbitrary starting position as long as the right sensor sees the right edge of the field. Under this working condition, the control goal is set to maintain the average longitudinal and lateral distances in the sensor frame with respect to the nearest crop equal to zero until the end of the row is reached. Afterwards, depending on the harvesting method, the harvester then either performs a $180^{o}$ turn and then engages the left sensor to follow the left edge of the field (cutting by go-and-return), or reverses the motion direction and drives backward until the vehicle is ready to perform a $90^{o}$ turn to re-engage the right sensor again with the right perpendicular edge of the field (oceanic cutting).

## 6. Simulation Results

In this section, simulation results are reported. In detail, the two tracking control techniques previously presented are compared and discussed. Then, the adoption of a safety overlapping distance in go-and-return cutting operation is discussed and analyzed considering the main irregularity parameters affecting its severity. Finally, a comparison between go-and-return and oceanic cutting strategies in terms of harvesting time and fuel consumption is reported.

### 6.1. Sensor vs. GPS-Based Control

**Figure 19:**
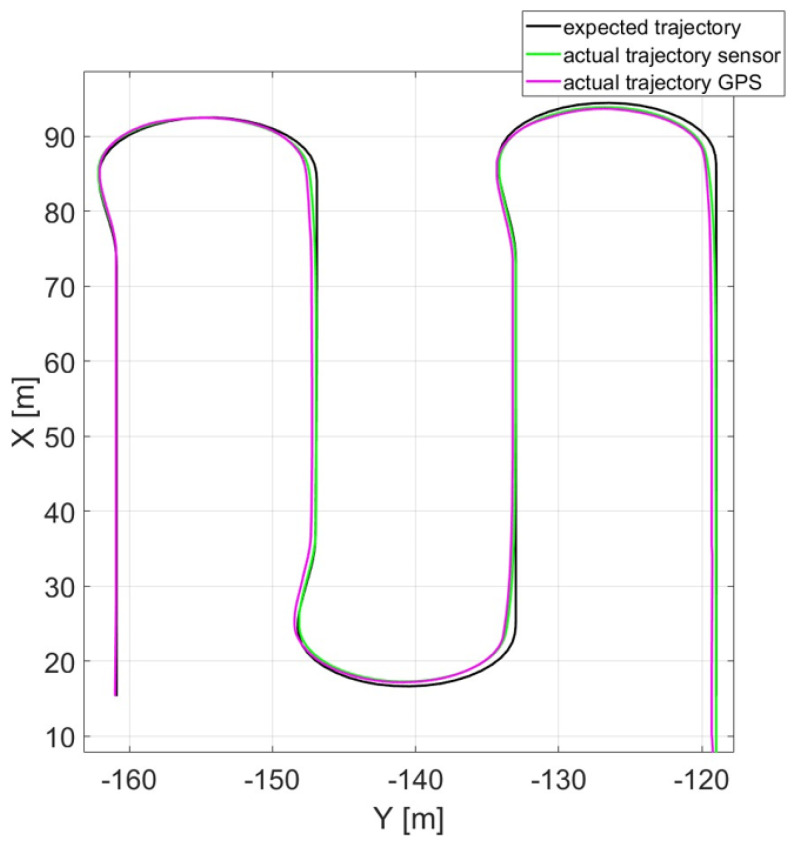
Expected trajectory against actual trajectories of sensor- and GPS-based control.

**Figure 20:**
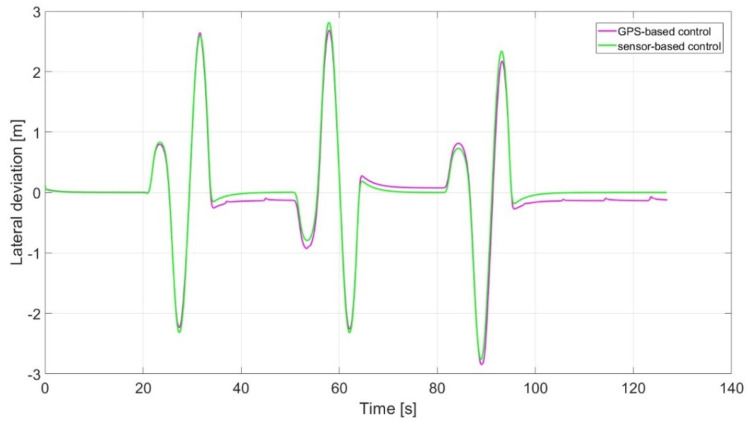
Lateral deviations from reference of sensor- and GPS-based control.

Simulation results highlight the advantages of vision-based control over the GPS-based one. In detail, vision-based control, even though it requires higher onboard computing power and the system’s ability to process sensor information in real-time, makes possible an online replanning of the trajectory without any a priori knowledge of field geometry that is required by a GPS-based controller. Moreover, its improved tracking performance reduces the need of overlapping over consecutive harvesting iterations. In Figure 19, the expected trajectory (black line) is compared to sensor-based (green) and GPS-based (purple) simulated trajectories. All trajectories are capable of following the reference, thus showing that a Stanley controller based on a point-by-point feedback is enough for operations inside the field (where cutting trajectory is usually almost rectilinear). A deeper comparison in terms of lateral deviation of the trajectories with respect to the reference one is reported in Figure 20.

The results show how the vision-based controller presents a lower tracking error (lateral deviation) when the harvesting operation is taking place (inside the field).

### 6.2. Overlap

**Figure 21:**
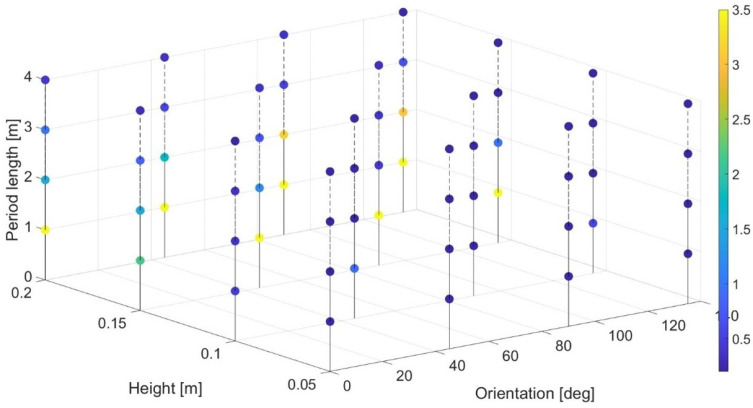
Effect of different wave parameter combinations on the required overlap.

In order to avoid missing crops during the harvesting operation, the adoption of a safe overlap distance is required, in particular when external disturbances (field irregularities) are present when applying a go-and-return technique. In real-life agricultural fields, irregularities in the terrain can vary widely; a common approach, also reproduced in simulation environment in CarMaker, is to consider the irregularity as a combination of three wave variables: orientation, height, and period length. Thereafter, an extensive simulation campaign constituted by 64 different scenarios—with all the possible combinations of the three wave variables where for each parameter a set of four different values is considered according to the state of the art in [17]—was performed to evaluate the need of a safety overlap distance for each scenario. In particular, the four values considered for orientation were 0°, 45°, 90°, and 135°, for the period length 1 m, 2 m, 3 m, and 4 m, and for the height 0.05 m, 0.1 m, 0.15 m, and 0.2 m. Figure 21 summarizes all simulation results. It is important to notice that in case an overlap is not necessary (no missed crops), a minimum safety distance of 0.2 m is suggested. On the other hand, in case of an unfeasible scenario (the vehicle fails in tracking the trajectory), an overlap of 3.5 m is reported (for clarity in the plots presented).

**Figure 22:**
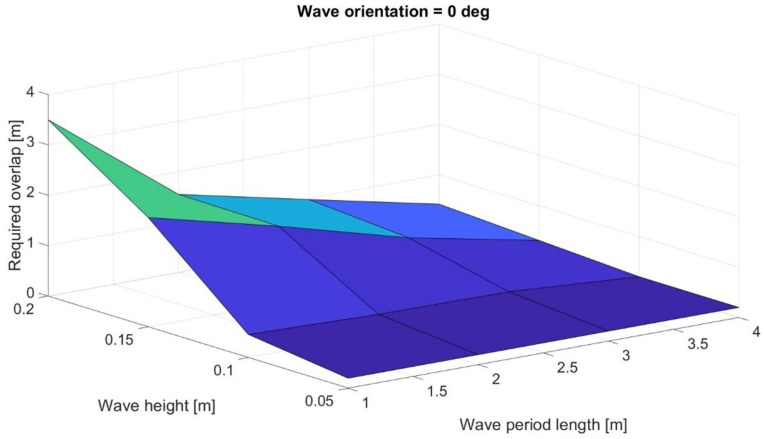
Effect of wave height and period length on the required overlap at zero-degree wave orientation.

From the simulation results it is possible to conclude that the larger the wave height the larger the overlap. At the same time, smaller wave length requires a larger overlap. Wave orientation influence is not clear. Therefore, the results of the previous simulation campaign can be reported in a figure in order to show overlap, wave height, and period length dependency considering a specific wave orientation to emphasize the effect of these two parameters. Figure 22, as an example, reports the case with zero degrees of orientation.

### 6.3. Go-and-Return vs. Oceanic Cutting

The second harvesting methodology considered in this study is oceanic cutting. It is characterized by following the borderlines of the field in a spiral motion until the whole area is covered. The two cutting strategies were tested and compared with one of the previously presented challenging scenarios where the required overlap of the go-and-return strategy was evaluated to be 1.55 m. Tracks of the cutter’s end line movement within the field is considered in order to compare the two strategies and to check whether the whole field was properly covered by the cutter.

**Figure 23:**
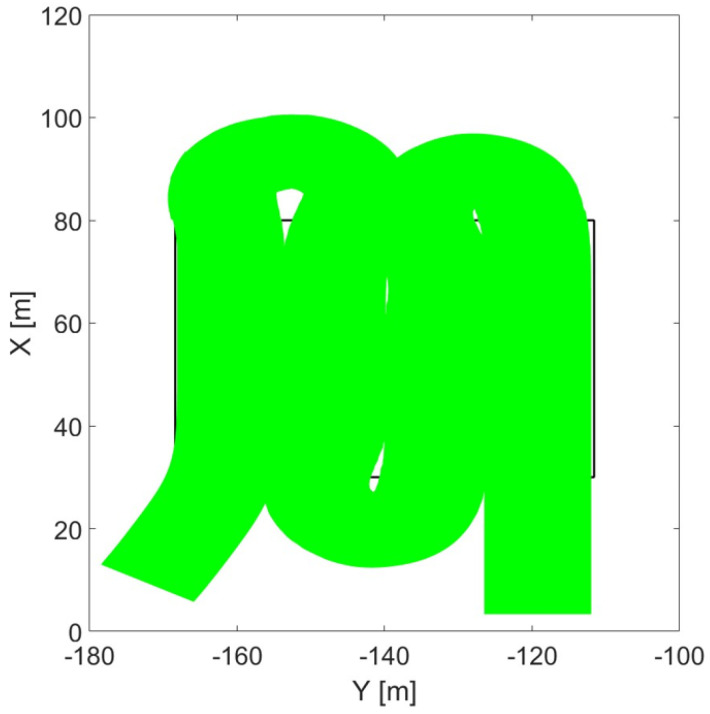
Cutter’s end line movement in cutting by go-and-return.

**Figure 24:**
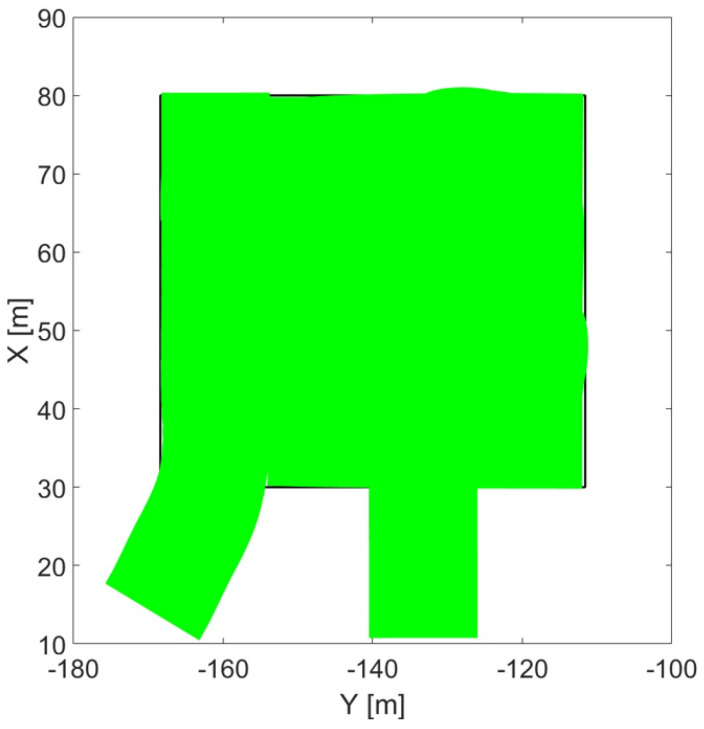
Cutter’s end line movement in oceanic cutting.

Figure 23, shows the cutter’s end line when cutting by go-and-return is performed and no overlap is implemented. The hsrvesting operation is performed in most of the area (green), but some uncovered (white) areas within the field borders (in black) are visible. This confirms the need of the adoption of an overlap to avoid missing crops. On the other hand, oceanic cutting simulation shown in Figure 24 demonstrated the self-recovering of the missed crops that would occur at the end of the first straight line. This proves how oceanic cutting does not need overlap adoption.

A comparison in terms of harvesting time between the two methods is provided in the following. In particular, considering a square field of a side length equal to 50 m and ideal road conditions (no irregularities), applying the go-and-return strategy required the vehicle four passes to complete cutting operation and considering a speed of 10 km/h, it took a total of 126.3 s. On the other side, applying an ocean cutting approach considering the same scenario and the same speed, it took 252.3 s. It can be seen that cutting by go-and-return is almost twice as fast as oceanic cutting; however, tests were repeated considering irregularities and so the need of safety overlap for go-and-return strategy. The safe overlap distance increased the number of required passes, making the overall harvesting time higher, whereas the oceanic cutting time only slightly increases due to pitch and/or roll oscillations, which affects the proper tracking control action a little. A sensitivity analysis of irregularities over the harvesting time is eventually conducted and the simulated results are reported in Table 1.

It can be noticed that harvesting time is not very sensitive to the severity of the field, but depends only on the number of required passes. It can be concluded that harvesting time is just affected by the overall number of passes required to complete the task. This means that for larger fields where the overlap can accumulate more and more passes in cutting by go-and-return, the oceanic cutting could become convenient.

As it can be deduced from the previous results, due to its variable speed and longer harvesting time, the oceanic cutting technique is more fuel-consuming than cutting by the go-and-return strategy whose speed remains mostly constant and harvesting time is shorter.

**Figure 25:**
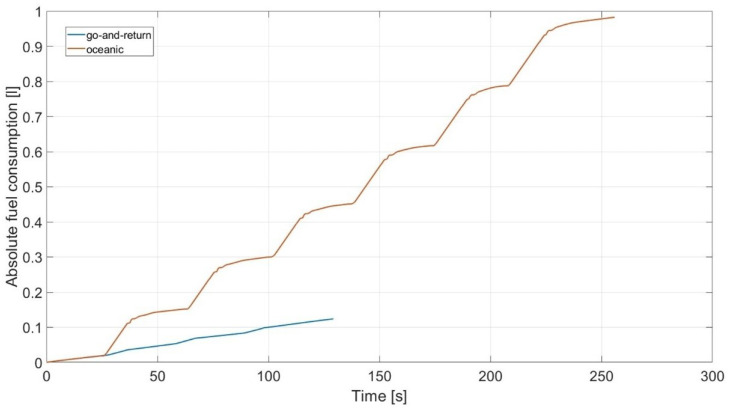
Consumption comparison between the two strategies.

Figure 25 shows the fuel consumption of the two strategies for the square field of a side length equal to 50 m and ideal road conditions already considered. Combine harvester consumption in cutting by go-and-return (blue line) is evaluated to be around 0.12 L of fuel to complete the operation, while it consumed almost 10 times more fuel (0.98 L) in oceanic cutting mode (red line), mainly due to the reversing motion maneuver. This can be seen clearly after the end of the first row of crops where the consumption of the two methods was the same. In fact, due to braking and motion reversing, oceanic cutting’s fuel consumption rose significantly at the end of every row/column.

Eventually, it is worth noticing that the oceanic cutting method does not allow a constant cutting speed like the go-and-return, thus worsening the ride comfort. On the other hand, the oceanic cutting does not require prior knowledge of the field since it consists of cutting by following the edge according to sensor feedback.

### 6.4. Driver-in-the-Loop (DIL)

**Figure 26:**
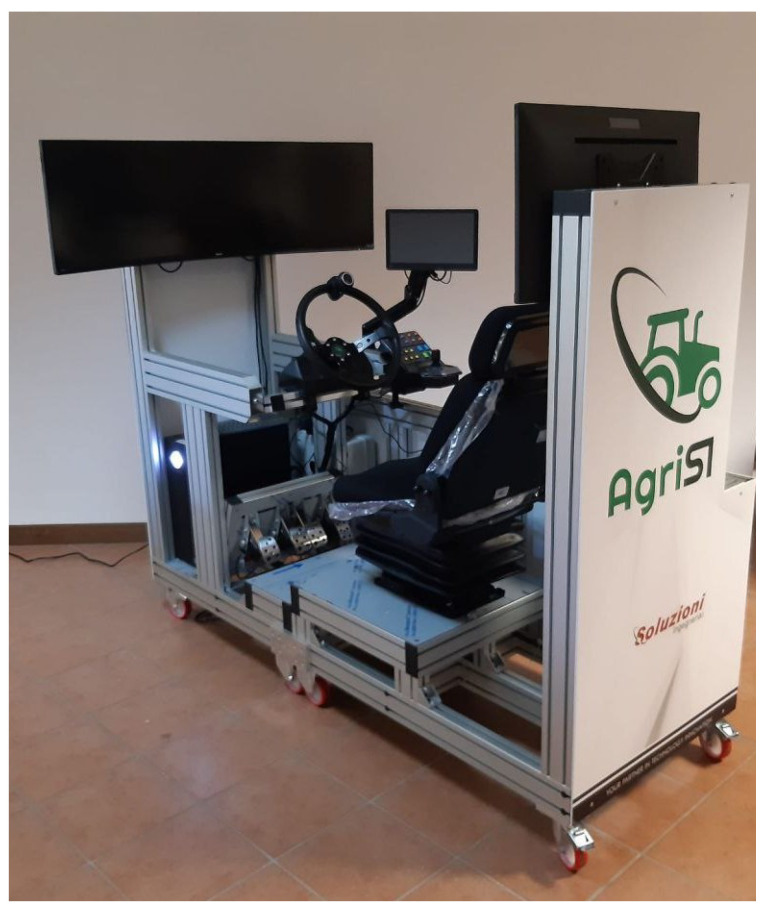
AgriSIm driving simulator.

In this section, a framework to compare human and autonomous driving is proposed: a Driver-in-the-loop approach based on a custom driver’s seat connected to the virtual simulator as shown in Figure 26. The experimental setup consists of a driving seat, all manual HMI commands for driving the vehicle, a display reporting the internal view of the driving cockpit, and the computer running the software. This system allows us to reproduce scenarios and harvester models as well as path planning resulting trajectories for the different harvesting strategies (as previously described). Thanks to an interactive interface, several Driver-in-the-loop (DIL) solutions to track trajectories are made available (manned, assisted, or unmanned).

This platform can be considered a reasonable methodology for a fair comparison considering the high repeatability of the test available and the fully controlled environment.

**Figure 27:**
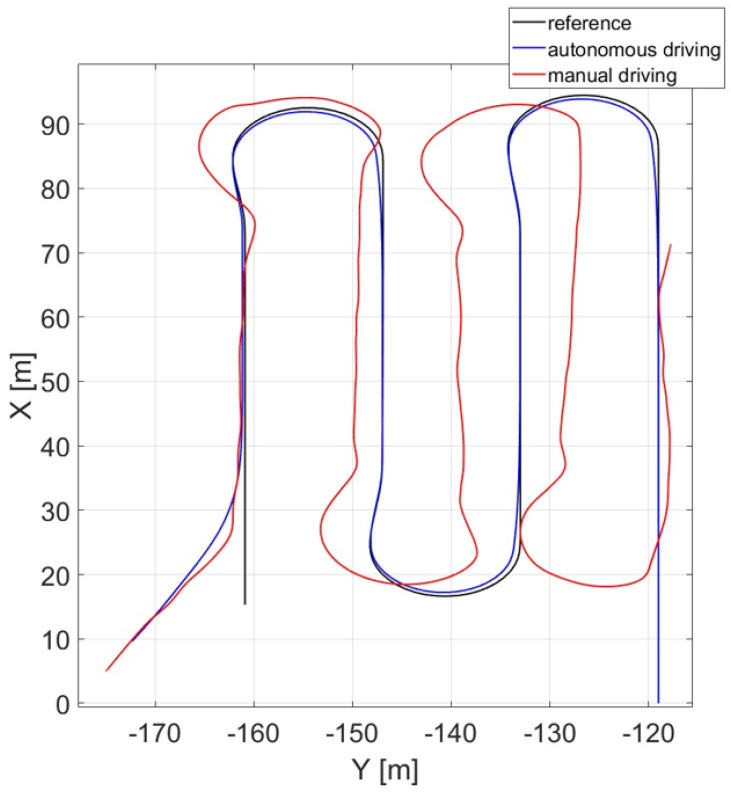
Reference trajectory tracking in autonomous vs. manual driving.

As an example, 10 attempts of manual driving were performed for cutting by go-and-return. In this paper, just the best human driven trajectory performed was reported (red line) and compared both to the automated one (blue line) and to the reference trajectory (black line) as shown in Figure 27.

As the human driver was not professional, the results cannot be considered as evidence of the advantages offered by an automated control over manual driving, even if from the figure manual driving appears to be more sensitive to irregularities and is not able to optimally turn and properly follow straight lines like automated driving.

As a future development, this methodology could be applied to perform a cost–benefit analysis of a control framework respect to a human-driven approach.

## 7. Conclusions

The presented research provides a full description of the virtual modeling and simulation of wheat crop, wheat field, and combine harvester. Proper path-planning and path-tracking algorithms were implemented.

In detail, two harvesting techniques were investigated and compared to assess their performance with the aim of reducing harvest losses and minimizing out-of-work time in the presence of ground irregularities simulated by means of sinusoidal waves. It was demonstrated how ground irregularities (high amplitude or low period) degrades cutting by go-and-return performances by requiring an higher overlap to avoid harvest losses. In these conditions the adoption of the slower oceanic cutting method (where no overlap is required) is preferable than the faster cutting by go-and-return. The path-tracking problem was then investigated and both a GPS-based and a vision-based implementation were presented. The Vision-guided Stanley Controller proved its effectiveness in following the crop edge even under the event of disturbances. The overall performance of the control shows that no abrupt maneuvers, potentially dangerous for the integrity of the crops, are required during inside-the-field operations due to the low speed. In order to test the developed control algorithms, co-simulations between CarMaker and Simulink were proposed.

Eventually, additional considerations and future development directions can be drawn:

- Operator Stress and Fatigue: this point could be investigated using the framework presented in Section 6.4 to show advantages of automated harvesting operation over human-driven ones;
- Soil Compaction and Passes: cutting by go-and-return, in absence of irregularities, guarantees better performances than oceanic cutting since the combine passes just once on each point of the field. In the presence of overlap, a deeper analysis could be conducted;
- Tire and Track Wear: cutting by go-and-return, in absence of irregularities, guarantees better performances than oceanic cutting since the combine travels a shorter distance. In presence of overlap a deeper analysis could be conducted;

## Figures and Tables

**Table 1:** Harvesting time as a function of field irregularities in cutting by go-and-return and oceanic cutting.

Irregularities			Go-&-Return		Oceanic
**Orientation (deg)**	**Height (m)**	**P. Length (m)**	**Overlap (m)**	**Time (s)**	**Time (s)**
No Irregularities			0	126.3	252.3
0	0.15	1	2.17	164.81	255.25
0	0.2	2	1.55	164.45	255.12
45	0.15	2	1.2	164.44	255.1
45	0.2	2	1.76	164.46	255.23
90	0.2	2	3.14	164.93	255.36
135	0.2	2	3.07	164.87	255.28

## Data Availability

Data are contained within the article.
